# Notes and News

**Published:** 1904-01-23

**Authors:** 


### NOTES AND NEWS.

MRS. EDWIN RICHARDSON has forwarded a cheque for £1,000 to the Sunderland Infirmary, to endow a bed in memory of her husband.

WE are asked to state that the annual meeting of the After-care Association for poor persons discharged recovered from asylums for the insane will be held at London House on Thursday, February 11th, at 3 P.M., and that the Bishop of London has kindly consented to preside.

THE farthing battle at Stratford-on-Avon, in which Miss Marie Corelli and Mr. Winter are taking part, is really very funny. Although Mr. Winter accepted the farthing awarded as damages to Miss Marie Corelli as the nucleus of a farthing fund for the local hospital, he will have none of her later contributions to swell his collection, which now amounts to 30,000 farthings.

IT has always been felt that whilst cremation is eminently desirable from a hygienic point of view, it opens up possibilities that the evidence of crime may pass undetected. A case occurred recently in Germany, where a doctor caused his two wives to be cremated, and after the death of the second one, and the destruction of the body, he was arrested on a suspicion of murdering her by poison.

FOR the safety of the public it is very evident that every care should be taken to secure men of the strictest integrity only as food inspectors, and that the emoluments are such as to attract men of a high standard of conduct. The temptations to which such officers are open was demonstrated quite recently when an inspector was offered £5 by a tradesman not to cause him to be prosecuted for the sale of margarine instead of butter. The temptation in such a case may be considerable because the officers may satisfy themselves that the fraud is not very great nor injurious to health, and a system of leniency may very easily become established.

THE Lambeth Guardians have appointed Miss N. V. Johnson as medical officer of their schools at West Norwood.

SIR R. DOUGLAS POWELL will open the winter course of lectures at the Hospital for Consumption, Brompton, on January 27th.

MRS. A. H. LEWIS has sent £2,500 to the King's Hospital Fund, which is the fourth quarterly subscription of the same amount which she has contributed.

WE have not yet learnt that the Metropolitan Asylums Board has received any offers for the purchase of the hospital ships *Atlas*, *Castalia*, and *Endymion*.

A MANIFESTO against juvenile cigarette smoking has been published by a large number of public men including all the head-masters of important schools, and a large number of medical officers and well-known practitioners.

THE seventh International Congress of Otology will be held at Bordeaux in August. During the congress the Lenval prize will be awarded for the best achievement in the treatment of aural affections or for the best apparatus for deafness.

WHILST the complaints about the bad weather of last year are universal, statistics show that the average of health was rather better than usual. From Leeds we learn that 1903 was a record healthy year, when the death-rate was only 16 6 of the population. The lower death-rate was especially noticeable in regard to zymotic diseases, as the deaths from infectious diseases showed an increase. The death-rate of Leeds was low, but that of London was lower still, amounting to only 15·7.

THIRTEEN theatres: Drury Lane, His Majesty's, Wyndham's, the New Theatre, the Lyric, Daly's, the Appollo, the Comedy, Strand, Terry's, Duke of York, Prince of Wales, and the Criterion, joined in a scheme suggested by the Theatrical Managers' Association, to hold a collection at a performance in to the new year to benefit the local hospital nearest the theatre—the result amounted to £288, in which Charing Cross and King's College Hospital participated. The Adelphi and the Vaudeville Theatre each sent a donation of £22.

AN interesting correspondence is being pursued in the *Times* on "Physical Deterioration." That "in the multitude of counsellors there is wisdom" is quite true in this connection, if the real grain of sense be extracted from the majority of these letters. The subject is complex and so is the remedy of the evil, for it must begin before an individual is born and continue until maturity. There is no more difficult education to conduct than that appertaining to health. Everyone is searching for a solution of the problem, and in the process the hospitals come in for a share of the blame. The old cry against the careful preservation of the unfit is once more raised

